# Revisiting the Windkessel Function: Toward Accessible Assessment of Central Arterial Health

**DOI:** 10.3390/jcm15010342

**Published:** 2026-01-02

**Authors:** Jun Sugawara

**Affiliations:** 1 Integrated Research Center for Self-Care Technology, National Institute of Advanced Industrial Science and Technology (AIST), Tsukuba 305-8566, Japan; jun.sugawara@aist.go.jp; 2 Faculty of Health and Sport Science, University of Tsukuba, Tsukuba 305-8574, Japan

**Keywords:** cardiovascular disease, arterial stiffness, risk assessment

## Abstract

Cardiovascular disease (CVD) remains the leading cause of death worldwide, accounting for nearly one-third of global mortality. Arterial stiffening, particularly in the central elastic arteries, impairs the Windkessel (cushioning and pumping) function and contributes to cardiovascular risk beyond traditional factors. Carotid–femoral pulse wave velocity (cfPWV) is established as the gold standard for assessing aortic stiffness and predicting cardiovascular and all-cause mortality; however, its technical complexity and requirement for trained personnel limit its use in routine clinical and community settings. These challenges have driven the development of simplified techniques for population screening, such as brachial–ankle PWV (baPWV). More recently, single-cuff oscillometric devices have emerged as practical alternatives. These methods are simple enough to be implemented in daily healthcare at home, thereby greatly enhancing accessibility, although their accuracy depends on model assumptions and calibration. In this perspective article, we highlight the pathophysiological significance of preserving the central arterial Windkessel function and emphasize the need for its practical assessment. Recent innovations mark a paradigm shift from complex laboratory-based measurements toward simplified, data-driven, and socially feasible screening tools for the early detection and prevention of CVD.

## 1. Introduction

Cardiovascular disease (CVD) remains the leading cause of death worldwide, accounting for nearly one-third of global mortality [1,2]. Despite major advances in pharmacological therapy and lifestyle modification, substantial residual risk persists even after optimal control of blood pressure, cholesterol, and glucose. A key contributor to this residual risk is arterial stiffening, particularly of the central elastic arteries.

Large elastic arteries such as the aorta and carotid arteries exhibit high distensibility. During systole, when blood is ejected from the left ventricle (LV), the wall of central elastic arteries stretches, temporarily storing part of the stroke volume within the lumen. This cushioning effect mitigates abrupt rises in pressure and reduces LV afterload [3]. During diastole, when ejection ceases, the stretched arterial walls recoil, acting as an auxiliary pump that propels the stored blood toward the periphery and prevents excessive declines in diastolic pressure. This mechanism facilitates coronary perfusion, which predominantly occurs in diastole, and transforms the intermittent LV output into a more continuous peripheral flow. Organs with low vascular resistance, such as the brain and kidneys, are particularly susceptible to pulsatile flow, yet they are protected by the cushioning and pumping actions of the central arteries [4]. The German physiologist Otto Frank described these functions as the “Windkessel” (air-chamber) effect [5]. Dr. Frank later formalized these mechanical principles in his two-element Windkessel model, which describes the elastic and resistive properties of the arterial system [5,6,7].

**Figure 1:**
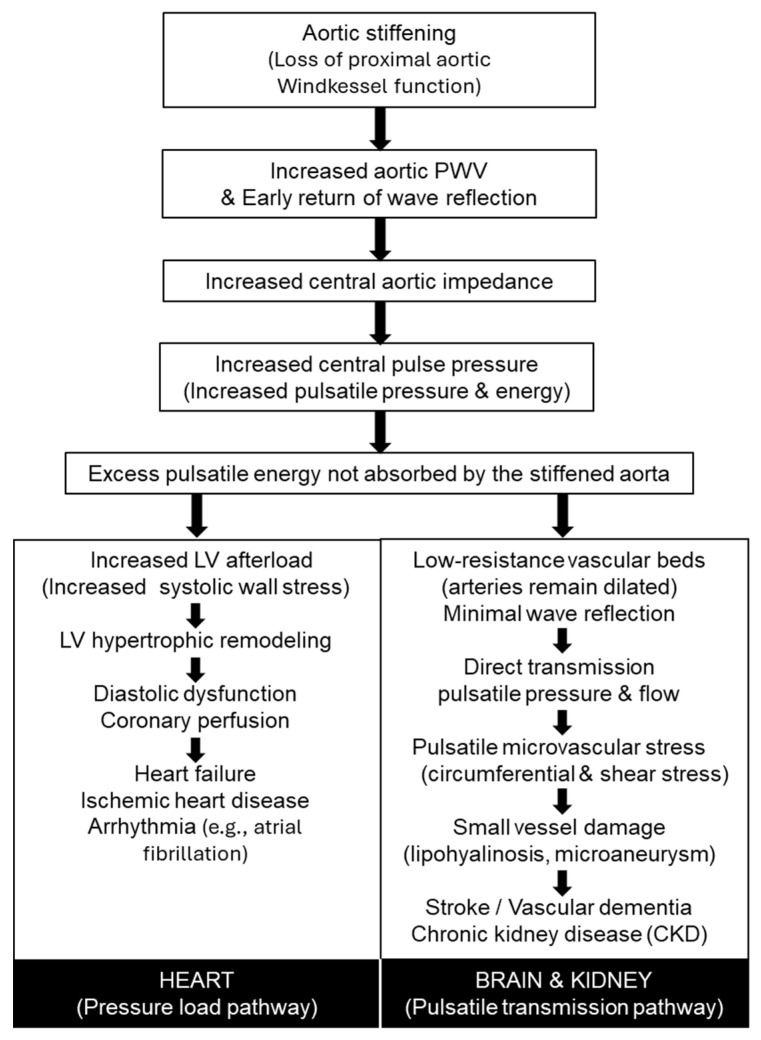
Pathophysiological pathways linking arterial stiffness to cardiovascular and end-organ disease (adapted from O’Rourke and Safar, 2005 [4]).

With advancing age, arterial stiffness increases, impairing the Windkessel function of the central arteries [8]. This impairment elevates LV afterload, reduces coronary perfusion, and amplifies pulsatile hemodynamic stress transmitted to vulnerable end organs such as the brain and kidneys [3]. Collectively, these effects explain why central arterial stiffening is a potent risk factor for CVD. Figure 1 summarizes the pathophysiological pathways linking increased arterial stiffness to cardiovascular and end-organ disease. Loss of proximal aortic Windkessel function leads to increased pulse wave velocity, early wave reflection, elevated central pulse pressure, and excess pulsatile energy that is no longer absorbed by the aorta. These central hemodynamic changes diverge into a pressure-load pathway affecting the heart—resulting in left ventricular remodeling, diastolic dysfunction, heart failure, ischemic heart disease, and arrhythmia—and a pulsatile transmission pathway affecting low-resistance organs such as the brain and kidneys, contributing to microvascular damage, stroke, vascular dementia, and chronic kidney disease.

## 2. cfPWV: The Gold Standard for Central Arterial Stiffness Evaluation

Pulse wave velocity (PWV) is a traditional yet the most well-established method for estimating arterial stiffness based on the speed at which the pulse wave travels through the arterial tree [9]. Since PWV is calculated from the pulse transit time between two distant arterial sites and the measured path length separating those sites, it can be measured at various locations throughout the body. However, accumulated evidence indicates that carotid–femoral PWV (cfPWV), which directly reflects the stiffness of the thoracic and abdominal aorta, is robustly associated with increased CVD events in both high-risk populations and community-based cohorts. A growing body of evidence supports cfPWV as the gold-standard index of central aortic stiffness and an independent predictor of total and cardiovascular mortality [10]. The European Society of Hypertension/European Society of Cardiology guidelines for the management of arterial hypertension suggested the measurement of aortic pulse wave velocity (PWV) as a tool for assessment of subclinical target organ damage [10]. Throughout a systematic review and meta-analysis, Vlachopoulos et al. demonstrated that each 1 m/s increase in cfPWV confers roughly a 10–15% higher risk of fatal and nonfatal cardiovascular events [11], underscoring the importance of aortic elasticity as a determinant of cardiovascular health. Major health organizations, including the European Society of Hypertension and the American Heart Association, recommend arterial stiffness assessment as a valuable adjunct for cardiovascular risk stratification [12,13]. Building on this framework, Bruno et al. recently demonstrated that cfPWV-derived vascular age captures clinically meaningful heterogeneity in arterial aging beyond chronological age [14]. By modeling vascular age from cfPWV together with traditional risk factors, they identified two biologically distinct phenotypes: Early Vascular Aging (EVA), characterized by disproportionately high arterial stiffness for age and a markedly elevated risk of cardiovascular events, and Supernormal Vascular Aging (SUPERNOVA), defined by unusually low stiffness and a substantially reduced risk. In a prospective cohort with 6.6-year follow-up, individuals classified as SUPERNOVA exhibited ~40% lower cardiovascular event risk, whereas those with EVA showed a ~2- to 3-fold higher risk, independent of conventional risk scores. These findings underscore the translational value of arterial stiffness as a biologically grounded marker of cardiovascular resilience or vulnerability.

However, widespread clinical adoption of cfPWV is limited by its technical complexity, dual-site measurement, and operator dependence. These barriers have motivated the development of simplified, non-invasive, and automated surrogate methods that could enable large-scale screening for early CVD risk detection.

## 3. baPWV and CAVI: The First Practical and Scalable Surrogate

Brachial–ankle pulse wave velocity (baPWV) represents the first successful translation of cfPWV into an accessible clinical and epidemiological tool [15]. The time component is derived from the difference between the pulse wave travel time from the heart to the ankle and that from the heart to the brachial artery, measured simultaneously using upper- and lower-limb cuffs. The distance component, representing the effective arterial path length, is estimated from the subject’s height. As a result, baPWV provides a composite index of systemic arterial stiffness that reflects the properties of both central elastic and peripheral muscular arteries.

In its early development, calculated PWV values often exceeded physiological ranges, prompting skepticism regarding which arterial segments were actually being assessed. This discrepancy largely arose from the use of height-based formulas to estimate path length [16], a source of systematic uncertainty that remains a practical limitation in contemporary baPWV measurements. Nevertheless, through the extensive accumulation of clinical evidence—mainly by Japanese investigators—the clinical and prognostic value of baPWV has been firmly established [17]. A meta-analysis of 18 cohort studies demonstrated that higher baPWV was associated with increased risks of total cardiovascular events, cardiovascular mortality, and all-cause mortality, with each 1 m/s increase corresponding to a graded rise in these risks [8,15]. Owing to its simplicity, operator independence, and high reproducibility, baPWV is widely regarded as a useful surrogate endpoint in clinical and epidemiological research and has become a cornerstone of vascular aging assessment in East Asian health screening programs [18].

In parallel with baPWV, the Cardio-Ankle Vascular Index (CAVI) was developed as an alternative stiffness metric designed to reduce the direct influence of blood pressure at the time of measurement [19]. CAVI incorporates the stiffness parameter β [20] and applies the Bramwell–Hill equation [21] to estimate arterial distensibility from the heart to the ankle. Accumulated evidence indicates that CAVI increases with age, correlates with cfPWV and baPWV, and predicts cardiovascular events, metabolic abnormalities, and progression of atherosclerosis [22,23]. Although CAVI was designed to minimize blood pressure dependence, it remains influenced by modeling assumptions inherent to the Bramwell–Hill relationship and may be susceptible to noise in β-stiffness parameter estimation.

Because of their extremely simple measurement procedures, both baPWV and CAVI have been widely adopted in clinical practice. However, their application remains largely confined to medical settings, and further innovation will be required to extend vascular aging assessment into community-based and population-wide screening contexts.

## 4. Surrogate Single-Cuff Oscillometric Devices: Simplifying Central Stiffness Assessment

A further step toward simplification came with single-cuff oscillometric systems that infer central stiffness from a single upper-arm recording [24,25,26]. A variety of single-cuff techniques have been developed to estimate central aortic hemodynamics from brachial pressure recordings, but they differ substantially in the type of waveform acquired and the analytical strategy used to reconstruct central pressure or derive stiffness indices.

One class of devices employs suprasystolic occlusion, inflating the cuff above systolic pressure to transiently halt brachial arterial flow [24]. Under complete occlusion, subtle wall-vibration signals can be recorded, containing forward and reflected wave components whose timing provides insight into arterial wave travel. Some systems derive a surrogate of aortic PWV by applying a simplified physical model to this reflection interval, together with reconstruction of the central waveform. Others analyze the same suprasystolic signal using more elaborate impedance-based modeling to separate forward and backward waves, producing central pressure and PWV-like indices that are more model-dependent than timing-dependent [25].

A second family of single-cuff techniques analyzes sub-diastolic oscillometric pulses collected during routine blood pressure measurement [26]. Here, central pressure is reconstructed using generalized transfer functions or arterial impedance models applied to the brachial oscillometric signal, and PWV estimates are derived via model inversion procedures that integrate waveform morphology with demographic and hemodynamic variables. These indices represent model-based estimates of vascular stiffness, rather than direct analogues of carotid–femoral transit time.

Together, these suprasystolic and oscillometric approaches illustrate the range of strategies currently used to extract central hemodynamic information from the brachial cuff. Recent validation studies have reported limited agreement between oscillometric pulse wave velocity and carotid–femoral pulse wave velocity, particularly at higher stiffness levels, underscoring that these measures should not be considered interchangeable [27]. Despite their differences, they share a common motivation: to enable practical, non-invasive assessment of arterial stiffness and wave reflection without relying on multi-site tonometry or Doppler-based transit-time methods.

## 5. Data-Driven Modeling of Vascular Aging

Within this broader landscape, several oscillometry-derived indices have been proposed to characterize arterial properties using only the brachial cuff signal. Among these, the Arterial Pressure–Volume Index (API) and the Arterial Velocity Pulse Index (AVI) serve as complementary measures that quantify the local pressure–volume relationship and waveform morphology of the brachial artery rather than reconstructing central waveforms [28]. Because these indices are derived from a peripheral muscular artery, not an elastic central artery, they are neither direct measures of aortic stiffness nor validated surrogate markers of it. Consequently, there is limited physiological rationale to assume that API or AVI reflects aortic functions such as the Windkessel properties of the proximal aorta. The underlying physiological determinants of these indices remain uncertain; therefore, elucidating factors such as arterial wall structural characteristics and the contribution of vascular smooth muscle tone will be essential for defining their physiological and clinical significance.

In this context, Ueno et al. recently proposed a data-driven vascular aging model using cuff-derived API and AVI analyzed with a generalized additive model [29]. This transparent, statistical-learning-based approach, distinct from autonomous “AI”, links vascular age to cardiac and renal dysfunction and offers an interpretable analytic framework for assessing vascular aging. A key advance was the adaptation of the vascular-age concept originally introduced by Bruno et al. [14]. Instead of relying on cfPWV, the central physiological marker in Bruno’s model, Ueno et al. substituted simple, cuff-derived indices (API and AVI) obtainable from a single upper-arm measurement.

However, an important conceptual distinction must be emphasized. Whereas Bruno’s model derives vascular age exclusively from cfPWV, an established marker of central arterial stiffness, Ueno et al.’s model incorporates API and AVI alongside multiple non-vascular predictors such as sex, chronic kidney disease (CKD) status, diastolic blood pressure, and heart rate. Notably, the regression coefficients for AVI and API are relatively small compared with those of the non-vascular factors, raising questions about the vascular specificity of the estimated age. Rather than capturing “pure” aortic aging, the model may instead reflect a broader constellation of systemic physiological burden, including renal dysfunction and hemodynamic alterations. Thus, the predicted vascular age represents a composite aging phenotype rather than a direct surrogate for central arterial stiffness.

Despite the conceptual limitations noted above, the clinical associations reported by Ueno et al. [29] are nonetheless informative. The difference between predicted vascular age and chronological age (ΔAge) showed clear relevance to subclinical organ function: individuals with accelerated vascular aging exhibited higher E/e′ ratios, elevated BNP concentrations, and reduced eGFR, linking the model to early cardiac and renal impairment [29]. These findings suggest that AVI/API-based vascular age may serve as an “integrated aging index” that aggregates multisystem physiological burden, even if it does not isolate vascular stiffness per se.

At the same time, several methodological issues require careful consideration. The retrospective, single-center design and partial dependence on blood pressure limit generalizability, and the absence of longitudinal outcome validation underscores the need for cautious interpretation. Future research should quantify the unique contribution of cuff-derived indices (e.g., standardized coefficients or partial R^2^), compare API/AVI-based vascular age directly with cfPWV-based models, and determine their prognostic value over time. Such analyses will clarify whether cuff-derived indices meaningfully capture vascular aging or primarily reflect overall health status.

Taken together, Ueno et al. [29] illustrate an important shift toward data-driven, clinically interpretable quantification of vascular aging. Their work demonstrates that simple cuff-derived indices can be integrated into a transparent statistical framework and linked to early cardiac and renal dysfunction, offering a pragmatic path for operationalizing vascular aging in preventive cardiology. Continued refinement—particularly through external validation, longitudinal outcome analyses, and clearer delineation of vascular versus non-vascular contributions—will determine how broadly such models can be adopted and how accurately they represent the biological progression of vascular aging.

## 6. Physiological Reinforcement: Heart-Brachium PWV

Many stiffness indices obtained from single-cuff oscillometric methods are derived from model-estimated aortic waveforms or local brachial arterial properties, rather than from direct measurements of central arterial stiffness. Given the pivotal role of proximal aortic Windkessel function in protecting the heart and vulnerable organs such as the brain and kidneys, there remains a strong need for simplified approaches that can reliably assess proximal aortic stiffness—or indices that directly reflect proximal aortic properties.

In this context, heart-brachium PWV (hbPWV) provides physiologically grounded reinforcement to these algorithm-based indices. hbPWV is calculated from the interval between the second heart sound and the dicrotic notch of the brachial pulse waveform, capturing pulse-wave propagation along a pathway that includes the ascending aorta and aortic arch before reaching the upper arm [30]. MRI-based validation studies demonstrated that hbPWV shows a pronounced age-related increase and correlates with central hemodynamic parameters, including aortic systolic pressure and augmentation indices, independent of brachial pressure [8]. These observations indicate that hbPWV primarily reflects functional stiffening of the proximal aorta.

Building on this physiological rationale, recent cross-sectional and longitudinal analyses further support the clinical relevance of hbPWV [31]. In more than 7800 adults, hbPWV displayed the steepest and most linear age-related rise among commonly used PWV measures, beginning as early as the early 30s. This contrasted with baPWV, which remained relatively flat until midlife and then accelerated abruptly thereafter. This pattern is consistent with early and progressive stiffening of the proximal aorta, which precedes marked changes in muscular arteries.

Notably, hbPWV showed stronger associations with the Framingham general CVD risk score than baPWV and maintained significant correlations even after adjustment for age and medication use. Over a 9-year follow-up, within-person increases in hbPWV tracked consistently with aging, whereas baPWV displayed more heterogeneous and nonlinear changes. Receiver-operating characteristic analyses further demonstrated that hbPWV better discriminated individuals with moderate-to-high CVD risk. Together, these findings suggest that hbPWV may complement established stiffness indices by capturing early functional changes in proximal aortic properties, rather than serving as a replacement for outcome-validated measures such as cfPWV.

**Figure 2:**
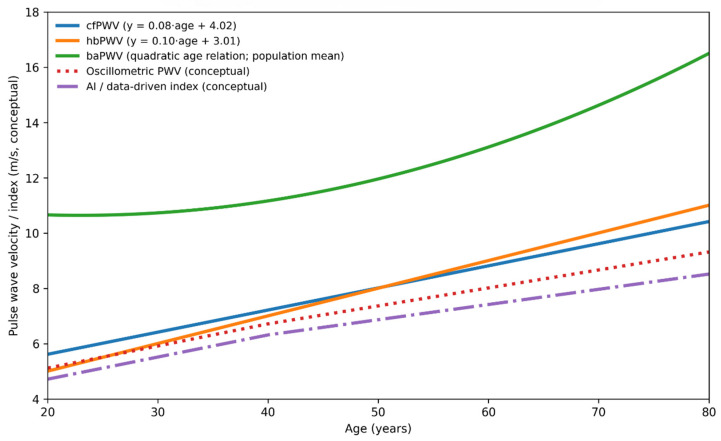
Conceptual age-related trajectories and validation differences across arterial stiffness indices (based on Sugawara et al. 2019 [8]; Yamashina et al. 2002 [15]; Prelević et al. 2024 [27]).

Figure 2 provides a conceptual comparison of age-related trajectories, physiological targets, and levels of outcome validation across arterial stiffness indices. Established measures, such as cfPWV and baPWV, demonstrate progressive age-related increases supported by longitudinal outcome data. In contrast, cuff-based oscillometric and data-driven indices follow more modest trajectories that reflect peripheral measurement characteristics and modeling assumptions rather than direct assessment of central aortic stiffness. This framework highlights that these indices should be interpreted as complementary, rather than interchangeable, tools for assessing vascular aging. To further summarize the physiological targets, strengths, limitations, and levels of clinical validation across established and emerging arterial stiffness indices, these approaches are compared in Table 1.

## 7. Other Indices

The Augmentation Index (AIx) represents a composite measure of arterial stiffness and the hemodynamic load imposed on the heart. Central arterial pressure waveforms are formed by the superposition of the primary forward pressure wave ejected from the left ventricle and the reflected waves that return from multiple peripheral sites. AIx is expressed as the proportion of pulse pressure attributable to this wave reflection [32]. When reflected waves return earlier, such as when aortic PWV is elevated, or when they are insufficiently damped by the peripheral vasculature, augmentation increases and AIx becomes higher. Thus, AIx partially reflects central arterial stiffness as well as peripheral vascular resistance.

AIx can be derived from carotid pressure waveforms measured using applanation tonometry or from radial arterial waveforms transformed into aortic waveforms using a generalized transfer function [32]. In addition, AIx can also be computed directly from peripheral arterial waveforms without reconstructing a central waveform [33]. Because all of these approaches historically relied on tonometry, skilled operator technique had been required. However, the recent introduction of oscillometric devices that estimate aortic pressure waveforms from brachial artery signals using air-plethysmographic cuffs has greatly simplified the measurement of aortic AIx [34].

It should be noted that AIx exhibits several characteristics distinct from cfPWV. A pronounced sex difference exists: cfPWV is typically lower in women than in men, whereas AIx is generally higher in women [35]. Furthermore, AIx increases with age until around 60 years, after which it tends to plateau [35]. These features clearly differ from the behavior of cfPWV. Nevertheless, central hemodynamic indexes, including AIx, are independent predictors of future CV events and all-cause mortality [11]. Recent meta-analytic evidence demonstrates that higher AIx was associated with higher LV mass index, independently of age, sex, presence of hypertension (or blood pressure level), and level of CVD risk [36].

As an even simpler index, the second derivative aging index obtained from the fingertip photoplethysmogram waveform (SDPTG) has been proposed, originally introduced by Takazawa and colleagues, offering an accessible means of assessing arterial wave reflection and vascular aging [37]. It is calculated from the ratios of the five characteristic inflection points of the acceleration plethysmogram. As arterial compliance declines with aging, these waveform components shift in a characteristic pattern, resulting in an increase in the SDPTG aging index. Thus, the SDPTG aging index serves as a simple, noninvasive indicator of vascular aging based on the contour of the peripheral pulse waveform. Indeed, SDPTG aging index in subjects with any history of diabetes mellitus, hypertension, hypercholesterolemia, and ischemic heart disease than in age-matched subjects without such a history [37]. Thus, it has the potential for evaluating arterial stiffness and wave reflection at the resting condition. However, it is easily affected by sympathetic stimulation, such as an arithmetic task, independent of arterial stiffness. Importantly, it needs to be measured under well-rested, relaxed conditions because the finger photoplethysmogram may easily be affected by a mental stressor [38].

## 8. Clinical Implications

The expanding availability of arterial stiffness-related indices, from cfPWV and baPWV to model-based indices, single-cuff oscillometric approaches, data-driven vascular-age metrics, and hbPWV, broadens opportunities for cardiovascular risk assessment beyond traditional hemodynamic measurements. As summarized in Table 1, these modalities differ substantially in physiological targets, feasibility, and levels of outcome validation, underscoring the need for complementary interpretation rather than reliance on a single metric. From a pathophysiological perspective (Figure 1), early stiffening of the proximal aorta compromises Windkessel function, increases central pulsatile load, and accelerates injury to both the heart and low-resistance organs such as the brain and kidneys; identifying this process before the development of overt hypertension or clinically apparent organ damage is therefore of central clinical importance. Consistent with the age-related trajectories illustrated in Figure 2, stiffness indices vary in their sensitivity to early versus later vascular aging: measures reflecting proximal aortic properties, including cfPWV and hbPWV, show relatively linear increases beginning in early adulthood, whereas more peripheral indices often accelerate later in life. In this context, hbPWV may help identify early functional stiffening of the proximal aorta, while remaining complementary to outcome-validated measures rather than serving as a replacement.

Simplified stiffness metrics also offer practical advantages for prevention and patient engagement. Automated and single-cuff approaches facilitate scalable screening, and vascular-age representations provide intuitive tools for communicating risk. However, as emphasized in Figure 2 and Table 1, differences in physiological targets and validation strength must be carefully considered to avoid overinterpretation. Taken together, integrating physiological insight (Figure 1), conceptual differentiation of indices (Figure 2), and systematic comparison of strengths and limitations (Table 1) provides a coherent framework for applying arterial stiffness assessment in clinical practice, with index selection best aligned to the clinical question, population, and stage of disease.

It should be recognized that arterial stiffness and vascular aging do not arise solely from arterial mechanics but reflect cumulative biological and social influences across the life course. Social determinants of health—including socioeconomic status, access to healthcare, and environmental exposures—modulate disease progression, biomarker expression, and outcomes. Accordingly, arterial stiffness indices should be interpreted not only as mechanical surrogates but also as integrative markers shaped by long-term physiological and social risk.

Social determinants of health—including socioeconomic status, access to healthcare, and environmental exposures—modulate disease progression, biomarker expression, and outcomes, as highlighted in a recent systematic review (Costa et al., 2023 [39]).

Finally, it should be recognized that arterial stiffness and vascular aging do not arise solely from arterial mechanics but reflect cumulative biological and social influences across the life course. Social determinants of health—including socioeconomic status, access to healthcare, and environmental exposures—modulate disease progression, biomarker expression, and clinical outcomes, as highlighted in a recent systematic review [39]. Accordingly, arterial stiffness indices should be interpreted not only as mechanical surrogates but also as integrative markers shaped by long-term physiological and social risk.

## 9. Conclusions

As global population aging accelerates, there is a pressing need for scalable, low-cost, noninvasive tools that extend vascular aging assessment beyond specialized clinical settings. Methods derived from routine cuff measurements, including data-driven vascular-age models and hbPWV, offer a pathway toward broad community-level implementation, requiring minimal technical expertise and integrating seamlessly into workplace, community, and population health screening.

These approaches represent a shift from resource-intensive diagnostics toward population-based prevention, enabling earlier identification of individuals at heightened vascular risk and supporting personalized, anticipatory cardiovascular care. By simplifying the assessment of central arterial aging and extending it to younger and middle-aged adults, these emerging technologies hold significant promise for reducing the societal burden of heart, brain, and kidney disease and promoting healthy longevity.

## Figures and Tables

**Table 1:** Organ-Specific Hemodynamic Consequences of Increased Aortic Stiffness: Heart Versus Brain and Kidney.

Index	Physiological Target	Strengths	Key Limitations	Outcome Evidence
cfPWV	Proximal aorta	Gold standard	Operator-dependent	✔✔✔
baPWV	Systemic	Scalable	Path-length error	✔✔
CAVI	Systemic	BP-independent	Model assumptions	✔✔
Oscillometric PWV	Model-based	Easy	Device variability	△
hbPWV	Proximal aorta	Early detection	Limited availability	✔

Abbreviations and validation levels are based on prior consensus statements and representative studies (Laurent et al., 2006 [10]; Vlachopoulos et al., 2010 [11]; Shirai et al., 2006 [19]; Ueno et al. [29]). Outcome evidence is graded as follows: ✔✔✔ = strong and consistent evidence; ✔✔ = moderate evidence; ✔ = limited evidence; △ = preliminary or inconsistent evidence.

## Data Availability

No new data were created or analyzed in this study. Data sharing is not applicable to this article.

## Abbreviations

The following abbreviations are used in this manuscript:

AI	artificial intelligence
AIx	augmentation index
API	arterial pressure–volume index
AVI	arterial velocity pulse index
baPWV	brachial–ankle pulse wave velocity
BNP	B-type natriuretic peptide
CAVI	cardio-ankle vascular index
cfPWV	carotid–femoral pulse wave velocity
CKD	chronic kidney disease
CVD	cardiovascular disease
E/e′	early diastolic mitral inflow velocity to mitral annular early diastolic velocity ratio
EVA	early vascular aging
eGFR	estimated glomerular filtration rate
hbPWV	heart-brachium pulse wave velocity
LV	left ventricle
SDPTG	the second derivative of the photoplethysmogram waveform
SUPERNOVA	supernormal vascular aging
